# Diagnosis of cervical lymph node metastasis with thyroid carcinoma by deep learning application to CT images

**DOI:** 10.3389/fonc.2023.1099104

**Published:** 2023-01-26

**Authors:** Tiantian Wang, Ding Yan, Zhaodi Liu, Lianxiang Xiao, Changhu Liang, Haotian Xin, Mengmeng Feng, Zijian Zhao, Yong Wang

**Affiliations:** ^1^ Department of Thyroid Surgery, the Second Affiliated Hospital of Zhejiang University College of Medicine, Hangzhou, China; ^2^ School of Control Science and Engineering, Shandong University, Jinan, China; ^3^ School of Medicine, Zhejiang University, Hangzhou, China; ^4^ Shandong Provincial Maternal and Child Health Care Hospital, Shandong University, Jinan, China; ^5^ Department of Radiology, Shandong Provincial Hospital Affiliated to Shandong First Medical University, Jinan, China; ^6^ Department of Radiology, Shandong Provincial Hospital, Shandong University, Jinan, China

**Keywords:** computer-aided diagnosis, deep learning, lymph node metastasis, computed tomography, neural network

## Abstract

**Introduction:**

The incidence of thyroid diseases has increased in recent years, and cervical lymph node metastasis (LNM) is considered an important risk factor for locoregional recurrence. This study aims to develop a deep learning-based computer-aided diagnosis (CAD) method to diagnose cervical LNM with thyroid carcinoma on computed tomography (CT) images.

**Methods:**

A new deep learning framework guided by the analysis of CT data for automated detection and classification of LNs on CT images is proposed. The presented CAD system consists of two stages. First, an improved region-based detection network is designed to learn pyramidal features for detecting small nodes at different feature scales. The region proposals are constrained by the prior knowledge of the size and shape distributions of real nodes. Then, a residual network with an attention module is proposed to perform the classification of LNs. The attention module helps to classify LNs in the fine-grained domain, improving the whole classification network performance.

**Results:**

A total of 574 axial CT images (including 676 lymph nodes: 103 benign and 573 malignant lymph nodes) were retrieved from 196 patients who underwent CT for surgical planning. For detection, the data set was randomly subdivided into a training set (70%) and a testing set (30%), where each CT image was expanded to 20 images by rotation, mirror image, changing brightness, and Gaussian noise. The extended data set included 11,480 CT images. The proposed detection method outperformed three other detection architectures (average precision of 80.3%). For classification, ROI of lymph node metastasis labeled by radiologists were used to train the classification network. The 676 lymph nodes were randomly divided into 70% of the training set (73 benign and 401 malignant lymph nodes) and 30% of the test set (30 benign and 172 malignant lymph nodes). The classification method showed superior performance over other state-of-the-art methods with an accuracy of 96%, true positive and negative rates of 98.8 and 80%, respectively. It outperformed radiologists with an area under the curve of 0.894.

**Discussion:**

The extensive experiments verify the high efficiency of the proposed method. It is considered instrumental in a clinical setting to diagnose cervical LNM with thyroid carcinoma using preoperative CT images. The future research can consider adding radiologists' experience and domain knowledge into the deep-learning based CAD method to make it more clinically significant.

**Conclusion:**

The extensive experiments verify the high efficiency of the proposed method. It is considered instrumental in a clinical setting to diagnose cervical LNM with thyroid carcinoma using preoperative CT images.

## 1 Introduction

The incidence of functional thyroid diseases has increased in recent years, and such diseases have become the second most common endocrine disorders (1). Although differentiated thyroid cancers have a good prognosis and low mortality rate, cervical lymph node metastasis (LNM) has been reported in 60–70% of patients and is considered an important risk factor for locoregional recurrence (2–7). Therefore, accurate preoperative diagnosis of cervical LNM in thyroid carcinoma is crucial for the proper selection of clinical treatment regimens and the prognosis of patients (8).

Although ultrasonography is considered the first choice for evaluating cervical LNM in thyroid cancer patients (9–12), it has not shown sufficient accuracy in the diagnosis of LNM in previous studies (13–15). Ultrasonography can only detect 20-31% of patients with central cervical LNM, whereas the detection rate for lateral cervical LNM is 70-93.8% (14, 15). Computed tomography (CT) is recommended for preoperative examinations of cervical LNM as an adjunct to ultrasonography in recent research and treatment guidelines (16–19). However, CT scans’ spatial resolution and contrast resolution are not high enough for cervical lymph nodes (LNs) to be accurately detected, as these LNs are not obvious and cannot be easily distinguished from accompanying blood vessels. Therefore, the diagnostic accuracies using CT depends on the level of radiologists, which puts less experienced radiologists at a greater risk of misdiagnosis or missed diagnosis. Especially for determining the surgical extent with cervical LNs on CT, undertreatment of metastatic neck nodes during primary surgery due to underdiagnosis will cause local recurrence, overtreatment with prophylactic lateral compartment dissection will increase surgical morbidity (10, 11).

Recently, deep learning techniques, especially convolutional neural networks (CNNs) (20), have successfully solved different classification tasks using CT images in the computer-aided diagnosis (CAD) domain (21–24). Following this trend, Lee et al. evaluated the performance of eight CNNs (ResNet50 performed best) in diagnosing cervical LNM on CT images (7) and compared the diagnostic performance with that of radiologists using an external validation set (25), which only proved the effectiveness of deep learning in diagnosing cervical LNM using CT images, but ignored an important clinic question of CAD: how to find LNs on the CT images by deep learning.

This study attempts to make the CAD process more consistent with radiologists’ diagnostic considerations by introducing a novel deep learning framework guided by the analysis of CT data for automated detection and classification of LNs in CT images. The proposed CAD framework consists of two main steps: (1) detecting the LN locations by an improved Faster R-CNN network; (2) classifying detected LNs with a residual network with an attention module. In the first step, an improved faster region-based convolutional neural network (Faster R-CNN) (26) is constructed to detect LNs at several scales, where lower-level feature maps are considered for small LN detection. Also, to further improve detection performance, the real distributions of LN size and shape are applied to design reliable anchors of each feature scale in the proposed detection network, leading to better detection. In the second step, the network integrating a residual network with an attention module is constructed to classify LNs. The attention module helps to classify LNs in the fine-grained domain, which leads to better performance of the whole classification network. The experimental results demonstrate that the proposed approach effectively diagnoses LNM with superior diagnostic performance than those of the three existing CAD methods and experienced radiologists.

## 2 Materials

**Figure 1:**
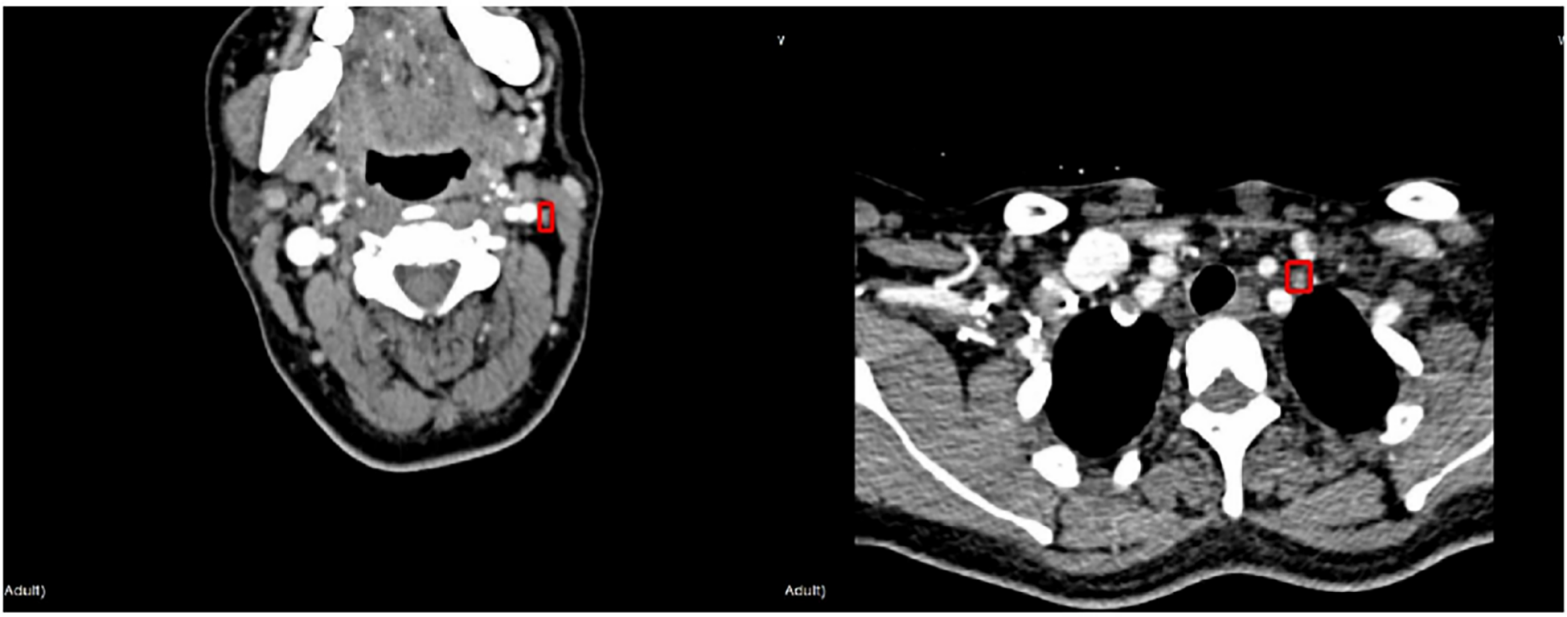
Illustration of typical LNMs: Benign case (left), malignant case (right).

**Figure 2:**
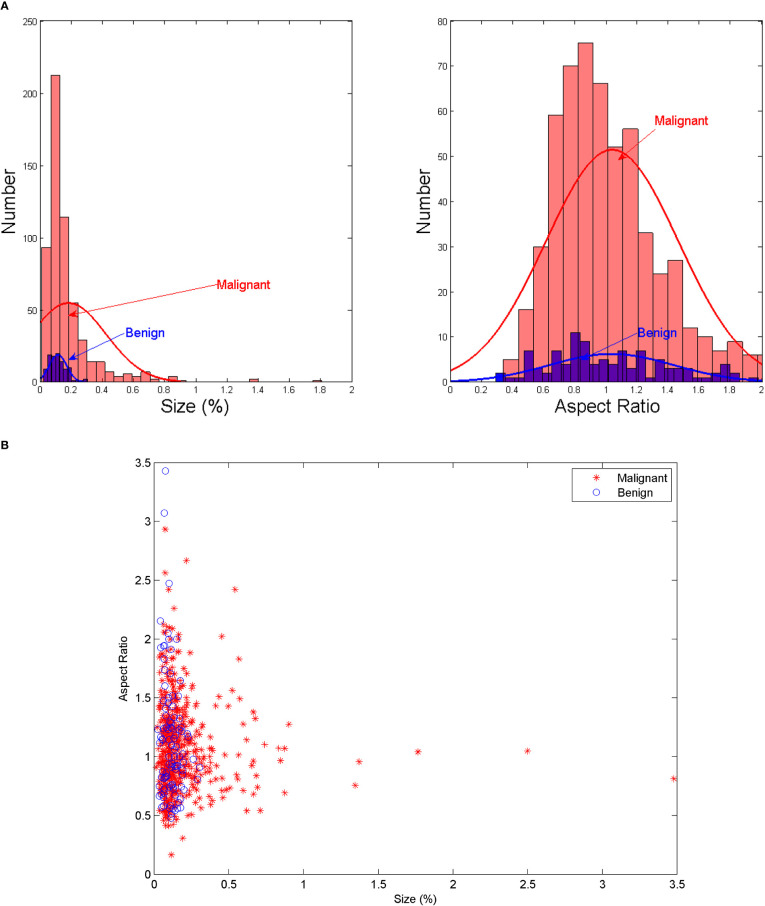
**(A)** The size and shape histograms of LNs **(B)** Joint distribution map of the size and shape of LNs.

The protocol of this retrospective study was approved by the Ethics Committee of the Institutional Review Committee of the Second Affiliated Hospital of the Zhejiang University, China. The patients who underwent CT examinations for surgical planning between January 2019 and June 2020 were prospectively recruited from a single institution, namely the Second Affiliated Hospital of Zhejiang University, China. All LNs included in the dataset were confirmed by fine-needle aspiration (FNA) and/or surgical pathology. A total of 574 axial CT images (including 676 lymph nodes: 103 benign and 573 malignant lymph nodes) were retrieved from 196 patients who underwent CT for the assessment as “suspicious” malignancy in the earlier examinations. Typical LNM CT images are shown in **Figure 1**. Compared with the whole CT image, the features in ROI area are more conducive to the judgment in the deep learning classification network. For each image, experienced radiologists draw the ground-truth region of interest (ROI) for LN detection, such as the red box in **Figure 1**. The dataset analysis shows that LNs (both benign and malignant) do not have a wide variety of sizes and shapes. The size is defined as the ratio of the LN area of the entire CT image; the width-to-height aspect ratio describes the shape. **Figure 2A** depicts the size and shape distributions of the LNs included in the adopted dataset. One can observe that most LNs are very small (size<0.5%) and fall into the aspect ratio domain of (0.5~2). The joint distribution map of the size and shape of LNs in the dataset is shown in **Figure 2B**, reflecting that benign and malignant LNs have a great overlap in size and shape. Therefore, it is difficult for radiologists to judge the benign and malignant LNs based on the size and shape information, so the image texture information of LNs in ROI is also required to simplify this task.

All CT images were obtained using 64 to 128 channel multi-detector CT scanners (SOMATOM Definition Flash, Siemens Healthineers; Brilliance, Philips Healthcare). Contrast-enhanced CT scanning was performed 40s after the intravenous injection of a 90-mL bolus of iodinated nonionic contrast material (300–350 mgI/mL) into the right arm, with a subsequent injection of a 20–30-mL saline flush at 3 mL/s using an automated injector. CT images were obtained with0.5–0.75 mm collimation and reconstructed into axial images every 2.0 mm on a 512×512 matrix using iterative reconstruction algorithms associated with each vendor’s CT scanner.

For detection, the 676 lymph nodes were randomly divided into 70% of the training set (73 benign and 401 malignant lymph nodes) and 30% of the testing set (30 benign and 172 malignant lymph nodes). Each CT image in the training and testing sets was expanded to 20 images by rotation, mirror image, changing brightness, and Gaussian noise. The extended data set included 11,480 CT images. For classification, ROI of lymph node metastasis labeled by radiologists were used to train the classification network. The training and testing sets for classification were set as same as the detection.

## 3 Methods

### 3.1 The combined detection and classification approach

**Figure 3:**
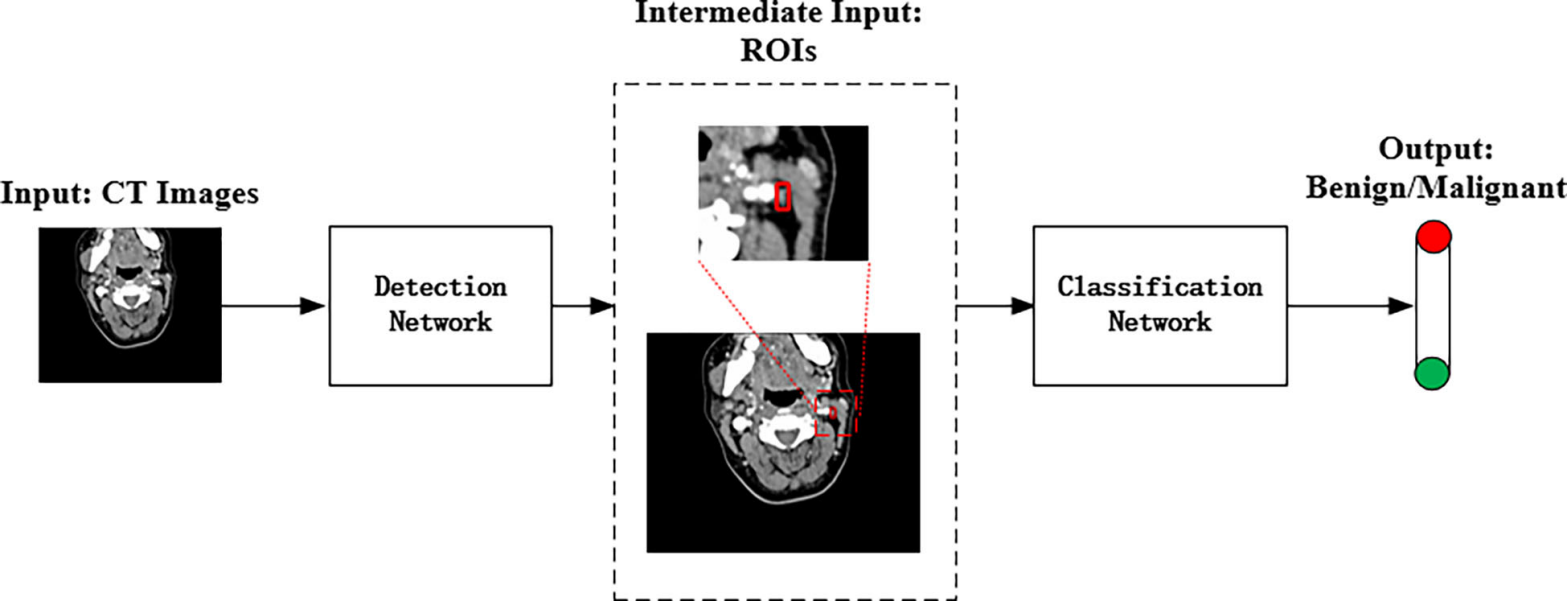
The pipeline of the proposed deep learning method for automated LN detection and classification.

The proposed method combined two deep convolutional networks to detect and diagnose LNs accurately. **Figure 3** shows the pipeline of the proposed method, where an improved Faster R-CNN was first designed to automatically locate LNs in CT images. Then, a residual network with an attention module was built to extract fine features for the LN classification.

#### 3.1.1 Detection network

**Figure 4:**
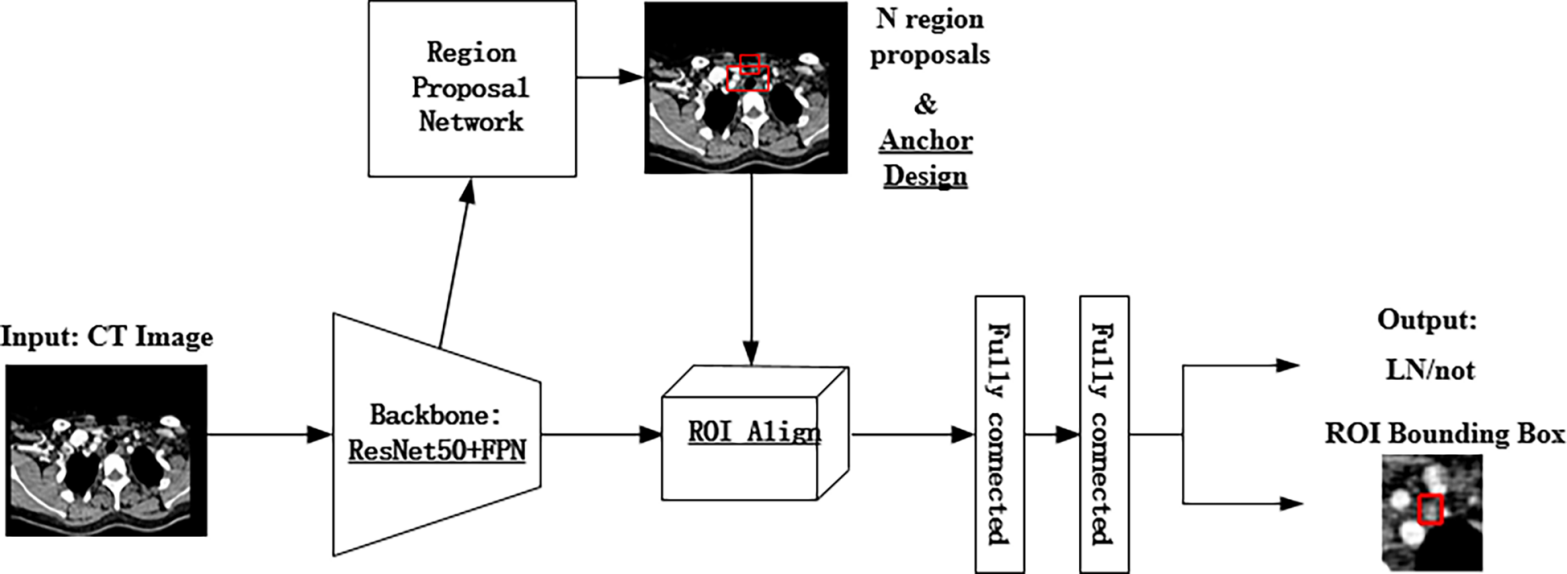
The pipeline of the improved Faster R-CNN.

According to **Figure 2**, the LN detection belongs to the category of small object detection. The Faster R-CNN has shown exciting performance in various object detection tasks (27–29). It comprises three modules: feature extraction layer, region proposal network (RPN), and classification layer. The Faster R-CNN is used to detect LNs, and the detection flowchart is shown in **Figure 4**. For a better adaptation of the system to the small-target detection task, the Faster R-CNN network was improved as follows: ResNet50+FPN (Feature Pyramid Network) was used to replace Visual Geometry Group (VGG) network as the extracted feature network; the region of interest (ROI) pooling (26) was replaced by the ROI align (30); the appropriate anchors were designed.

**Figure 5:**
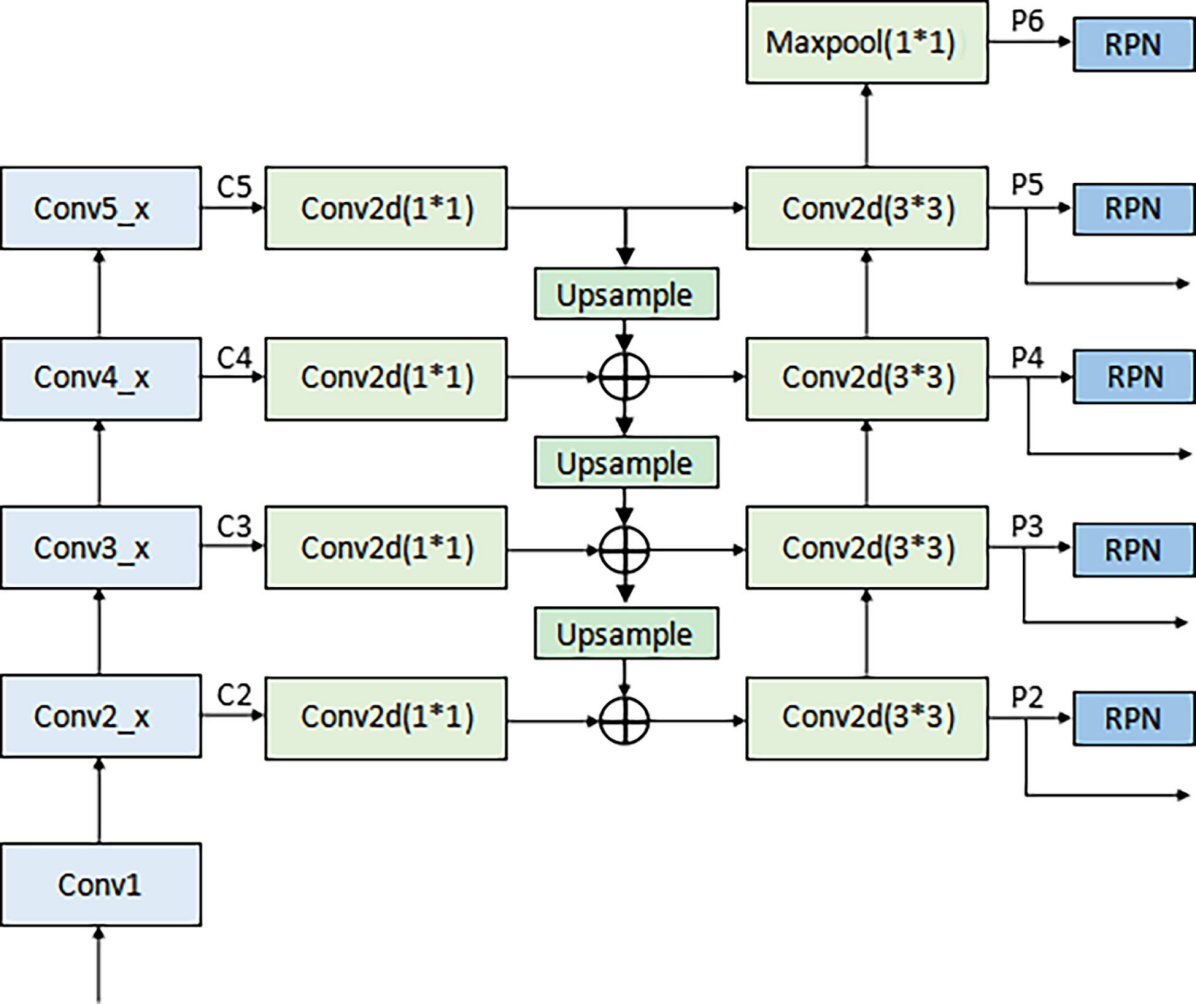
The architecture of backbone network in Faster R-CNN (ResNet50+ FPN).

A) Backbone network. Although ResNet50 (31) alleviates the problems of difficult network training and reduced performance caused by the deepening of the network, making its high-level features rich in semantic information, their low resolution is not conducive to detecting small-target objects. To solve this problem, the FPN proposed by Lin et al. (32) was adopted to fuse the feature from low to high levels and improve the detection precision of the model. The architecture of the backbone network (ResNet50+ FPN) in Faster R-CNN is shown in **Figure 5**. A 1×1 convolutional layer was attached to C2, C3, C4, and C5 (the feature activation of conv2, conv3, conv4, and conv5 outputs of ResNet50), and then the spatial resolution was upsampled by a factor of 2 using the nearest neighbor upsampling. The upsampled map was then merged with the corresponding bottom-up map by the element-wise addition. Finally, a 3×3 convolution was appended on each merged map to generate the final feature map. Multi-scale feature maps (P2, P3, P4, and P5) need to be input into the RPN network to generate candidate boxes and serve as the input part of classification and regression operation in the second stage. The feature map (P6) generated from the feature map of the topmost layer of ResNet50 after the maximum pooling was only used as the input part of RPN. The function of RPN is to combine the prior anchors to classify the background and foreground areas. After classification, a large number of prior anchors are screened out, making the anchors closer to the real target. In the RPN, the public Feature Map of Faster RCNN is processed by sliding window, which can also be regarded as a 3x3 convolution operation on the feature map, and then two full connection operations are performed on each feature vector, one gets 2 scores, and one gets 4 coordinates, and then combined with the predefined anchors, the candidate anchors is obtained after processing.

B) Input of the RPN. The full name of RPN is Region Proposal Network, which is called region generation network. It is a classless object detector that calculates regional targets through sliding windows. The input is an image of any scale, and the output is a series of rectangular candidate regions.

C) ROI Align. ROI pooling was used in the original Faster R-CNN network to convert ROI features of different sizes into the same feature map. In this process, the calculation results of coordinates were rounded twice: (i) when the region proposal when mapped to the shared feature map and (ii) when the feature map was fixed to a unified size. These round operations introduced misalignments between the ROI and the extracted features, which harmed the predictive accuracy of the small-target location. To solve this problem, ROI Align was used instead of ROI Pooling. RoI Pooling can intercept the feature of each Region of Interest in the feature map, and replace it with the feature output of the same size. Each quantization operation corresponds to a slight misalignment of regional features, and these quantization operations introduce bias between RoI and extracted features. The core idea of RoI Align is bilinear interpolation. The bilinear interpolation algorithm makes full use of the four real pixel values around the virtual point in the source image to jointly determine a pixel value in the target image. For a destination pixel, set the floating point coordinates obtained by inverse transformation. The coordinates are (i+u,j+v) (where i and j are the integer parts of floating-point coordinates, and u and v are the fractional parts of floating-point coordinates, which are floating-point numbers in the range [0,1)], Then the value of this pixel f(i+u,j+v) can be obtained from the coordinates in the original image as (i,j), (i+1,j), (i,j+1), (i+1,j+ 1) The value of the corresponding four surrounding pixels is determined. If the fixed feature graph had a small size, bilinear interpolation 33 and floating-point number were used to record the coordinate results, which improved the accuracy of locating small targets.

D) Anchor design. Anchor is the core of RPN network, and the generation of anchor without convolution operation, which is equivalent to sliding window calculation. After the image is input, Windows of different sizes are obtained by calculating the proportion of the sliding window center to the target size, length, width and multiple, which is called the basic standard window, through several stride, the map is reduced by many times. When the image is convolved at the last layer, the pixel will be reduced to the corresponding multiple, and tens of thousands can be obtained through the sliding window mapping to the input image based on the basic standard window of the output image. The preliminary selection of anchors in the original Faster R-CNN network was manually performed. The aspect ratio distribution in terms of the LN size on the dataset was pre-computed with the results shown in **Figure 2**. As seen in **Figure 2**, the LN aspect ratio roughly ranged from 0.5 to 2, and the LN size mainly ranged from 0 to 0.2%. This implies that the aspect ratio and size of anchors and proposed regions should also be selected within these ranges. Therefore, the anchors were assigned the areas of 256, 576, 1282, 1600, 3136 pixels (corresponding to the LN size of 0.01, 0.03, 0.06, 0.08, and 0.15%) on feature maps P2, P3, P4, P5, and P6 respectively. Besides, anchors of multiple aspect ratios (1:2, 1:1, 2:1) were used at each level. This amounted to the total number of fifteen 15 anchors in the FPN.

The loss function of Faster R-CNN can be roughly divided into two parts: multi-task loss of RPN and multi-task loss of Fast R-CNN. When training RPN network, the loss function of a picture is defined as:


$$L\left(\left\{p_{i}\right\},\left\{t_{i}\right\}\right)=\frac{1}{N_{cls}}\sum_{i}L_{cls}\left(p_{i},p_{i}^{*}\right)+\lambda \frac{1}{N_{reg}}\sum_{i}p_{i}^{*}L_{reg}\left(t_{i},t_{i}^{*}\right)$$

where *L_cls_* is the classification loss, *L_reg_* is the bounding box regression loss. *p_i_* is the probability that the ith anchor is the target object; p_i_* is the real label. *t_i_* is the boundary box regression parameter for predicting the ith anchor. t_i_* is the regression parameter of the real box corresponding to the ith anchor. *N_cls_* is the number of all samples in a small batch; *N_reg_* is the number of anchor positions; The default value of *λ* is 10. The loss function of Fast R-CNN is defined as:


$$L\left(p,u,t^{u},v\right)=L_{cls}\left(p,u\right)+\lambda \left[u\geq 1\right]L_{loc}\left(t^{u},v\right)$$

where *L_cls_* is the classification loss, *L_loc_* is the bounding box regression loss. *p* is the Softmax probability distribution predicted by classifier. *u* is the corresponding target real category tag; [*u*≥1] indicates that the value is 1 when *u*≥1, and 0 in other cases. *t^u^* is the regression parameter of the corresponding category *u* predicted by the boundary box regression; *v* is the bounding box regression parameter of the real target.

#### 3.1.2 Classification network

After detecting the location of LNMs by the improved Faster R-CNN network, we feed the detected ROIs into our attention-based classification network for LNM fine-grained classification.

ResNet50 was selected as the backbone network for the proposed model due to its excellent performance in diagnosing cervical LNM reported in the earlier study (7). The initial ResNet50 introduced by He et al. (31) consisted of one convolutional layer (Conv1) and four residual modules (Conv2_x to Conv5_x). A max-pooling layer followed each of them to downsample the feature maps by a scale factor of 2. Conv2_x to Conv5_x had 3, 4, 6, and 3 bottleneck blocks, respectively.

**Figure 6:**
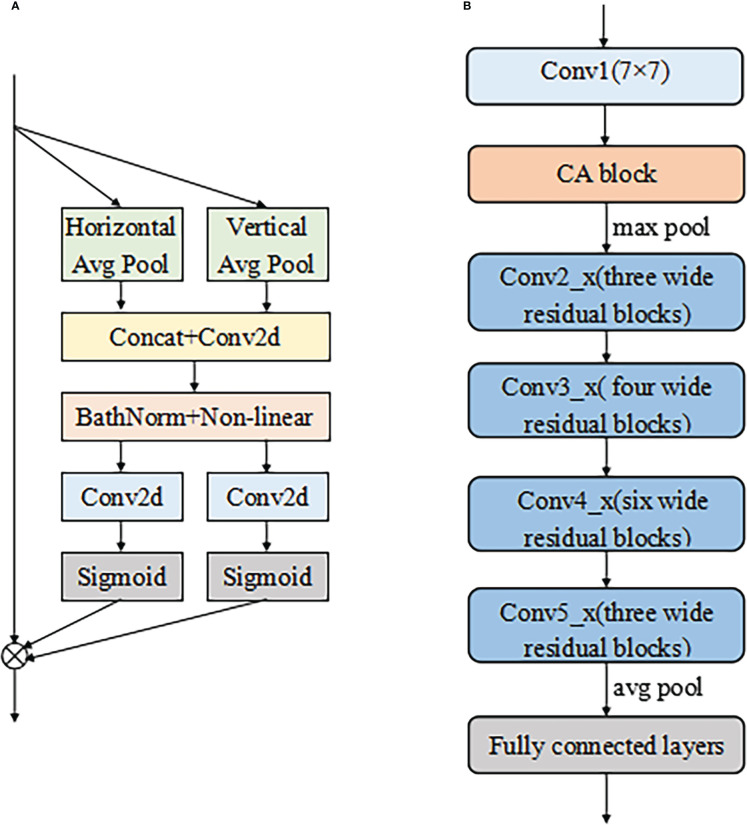
The structure of CA module **(A)** and the proposed A-ResNet50-W model **(B)**. Here the symbol ⊗ represents multiplicatio.

According to **Figure 2B**, the differentiation of benign and malignant LNs is not obvious. For better fine-grained classification, a coordinate attention (CA) module (33) was incorporated in the proposed model, as shown in **Figure 6A**. This module made it possible to focus on important texture features and suppress unnecessary ones. The CA encoded both channel relationships and long-range dependencies with precise positional information in two steps: (i) coordinate information embedding and (ii) CA generation. Moreover, wide residual blocks were developed by setting twice the number of channels of the initial ResNet50 in each bottleneck block to improve the classification performance, referring to the previous study (34). The refined model was intuitively named A-ResNet50-W (A standing for attention module and W for wide residual block). The proposed model structure is shown in **Figure 6B**.The convolution layer and output image size of Resnet50 at each stage are shown in **Table 1**.

**Table 1:** Convolution layer and output image size of Resnet50 at each stage.

Layer name	Output size	stage
Conv1	112×112	7×7, 64, stride 2
Conv2_x	56×56	3×3 max pool, stride 2 $\left[\begin{array}{c} 1\times 1,\;64\\ 3\times 3,\;64\\ 1\times 1,\;256 \end{array}\right]$ ×3
Conv3_x	28×28	$\left[\begin{array}{c} 1\times 1,\;64\\ 3\times 3,\;64\\ 1\times 1,\;256 \end{array}\right]$ ×3
Conv4_x	14×14	$\left[\begin{array}{c} 1\times 1,\;64\\ 3\times 3,\;64\\ 1\times 1,\;256 \end{array}\right]$ ×3
Conv5_x	7×7	$\left[\begin{array}{c} 1\times 1,\;64\\ 3\times 3,\;64\\ 1\times 1,\;256 \end{array}\right]$ ×3
	1×1	Average pool

### 3.2 Performance and statistics

Firstly, the automated detection performance of the proposed detection network was assessed and compared with that of three state-of-the-art detection methods. Next, the LN recognition performance of the proposed classification network was evaluated and subjected to a comparative analysis against several state-of-the-art classification methods. Finally, the developed networks trained on the proposed dataset were used to classify testing images. Their classification results were compared with the diagnoses of three senior radiologists with six to ten years of clinical experience on LNM diagnosis. The radiologists were unaware of the final diagnosis and the deep learning analysis results. Their “blind reviews” were based on the same image viewer, with freely scrollable images and adjustable window level/width. Finally, the radiologists identified these CT images as benign or malignant LNMs.

#### 3.2.1 Detection results

Ablation experiments of Faster R-CNN were conducted with four structures: (i) ResNet50, (ii) ResNet50 + FPN, (iii) ResNet50 + FPN + ROI Align, and (iv) ResNet50 + FPN + ROI Align + Anchor Design. The latter structure was proposed in this study. The detection performances are summarized in **Table 2**. The average precision (denoted as AP_50_) was used to quantitatively evaluate the detection performance, with the Intersection over Union (IoU) threshold of 0.5. As shown in **Table 1**, the proposed structure for the Faster R-CNN achieved the best AP_50_ and was considered suitable for detecting small LNs.

**Table 2:** Detection performance of different structures.

Faster R-CNN structure	AP50
ResNet50	72.
ResNet50+FPN	74.5
ResNet50+FPN+ROI Align	77.6
ResNet50+FPN+ROI Align+Anchor Design	80.3

**Figure 7:**
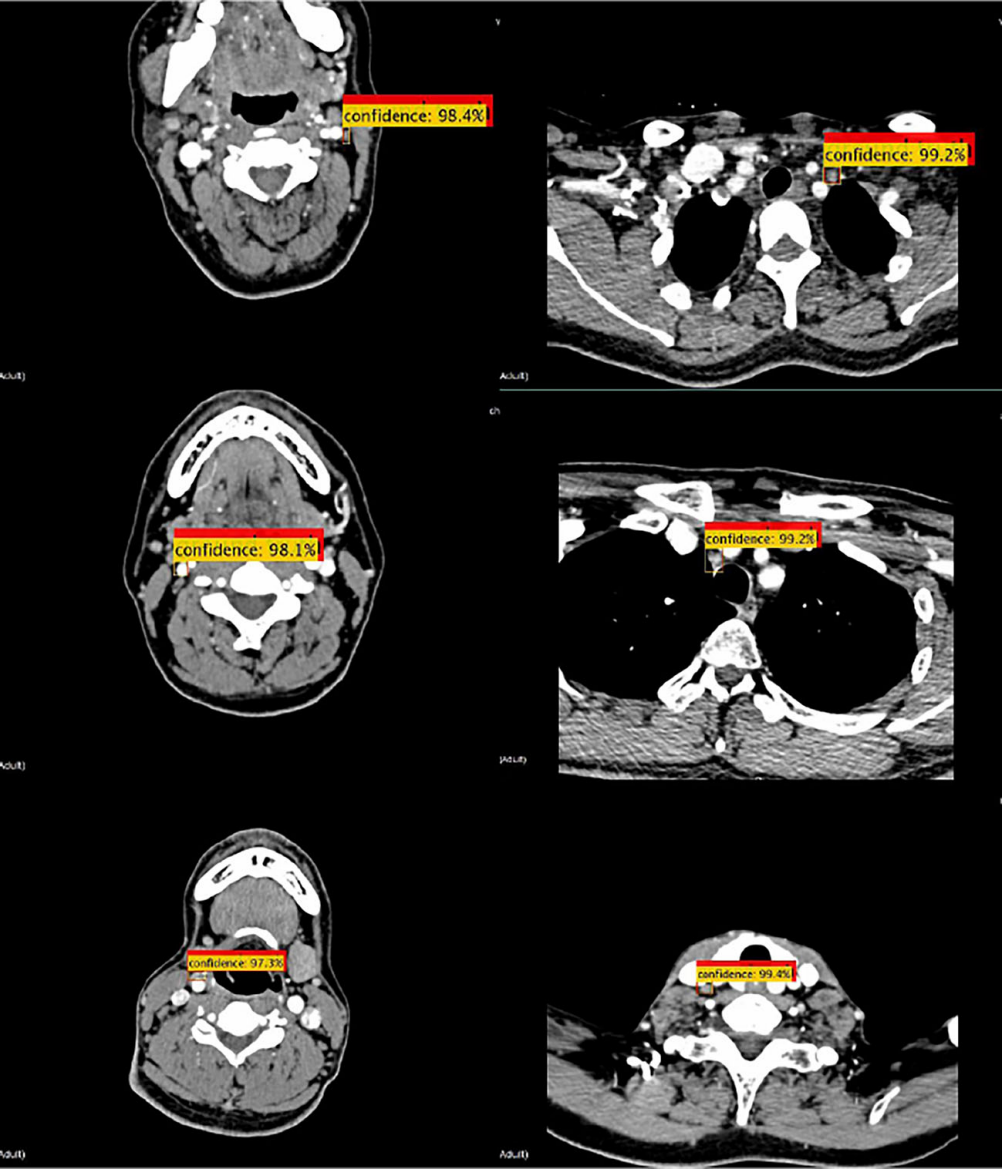
Detection samples of the proposed method. Red boxes illustrate the ground truth ROIs, while yellow boxes illustrate the successfully detected LNs and their confidence.

Some convolutional neural networks have been successfully applied to detection tasks, exhibiting a good performance. In this study, the proposed method was compared with three state-of-the-art neural networks, namely Faster R-CNN(ResNet50) (26), SSD (35), and Yolov3 (36). The detection samples of the proposed method are shown in **Figure 7**. The AP_50_ values of detection by the four methods are listed in **Table 3**, which indicates that the proposed detection method outperformed all others in AP_50_ over the other three methods by 2-4%.

**Table 3:** Detection performance in different networks for the testing set.

Network	AP50	Difference in AP50, %
Faster R-CNN (ResNet50)	77.9	2.4
SSD (ResNet50)	72.5	7.8
Yolov3 (DarkNet53)	76.2	4.1
The proposed method	80.3	–

#### 3.2.2 Classification results

The ablation experiments were performed on the same dataset with four structures: (i) a reference ResNet50 structure, (ii) the proposed structure A-ResNet50-W with the CA block (A) and the wide residual block (W), (iii) the proposed structure without the CA block (ResNet50-W), and (iv) the proposed structure without the wide residual block (A-ResNet50). The classification performances are summarized in **Table 3**. Quite naturally, the proposed structure (i.e., A-ResNet50-W) outperformed its “light versions” and the original ResNet50 network by ACC and TPR parameters. Insofar as both A-ResNet50 and ResNet50-W also surpassed the ResNet50 in ACC and TPR values, the CA and wide residual blocks were instrumental in the classification task execution. Meanwhile, TNR values of the proposed and ResNet50 networks were the same (80.0%).

**Figure 8:**
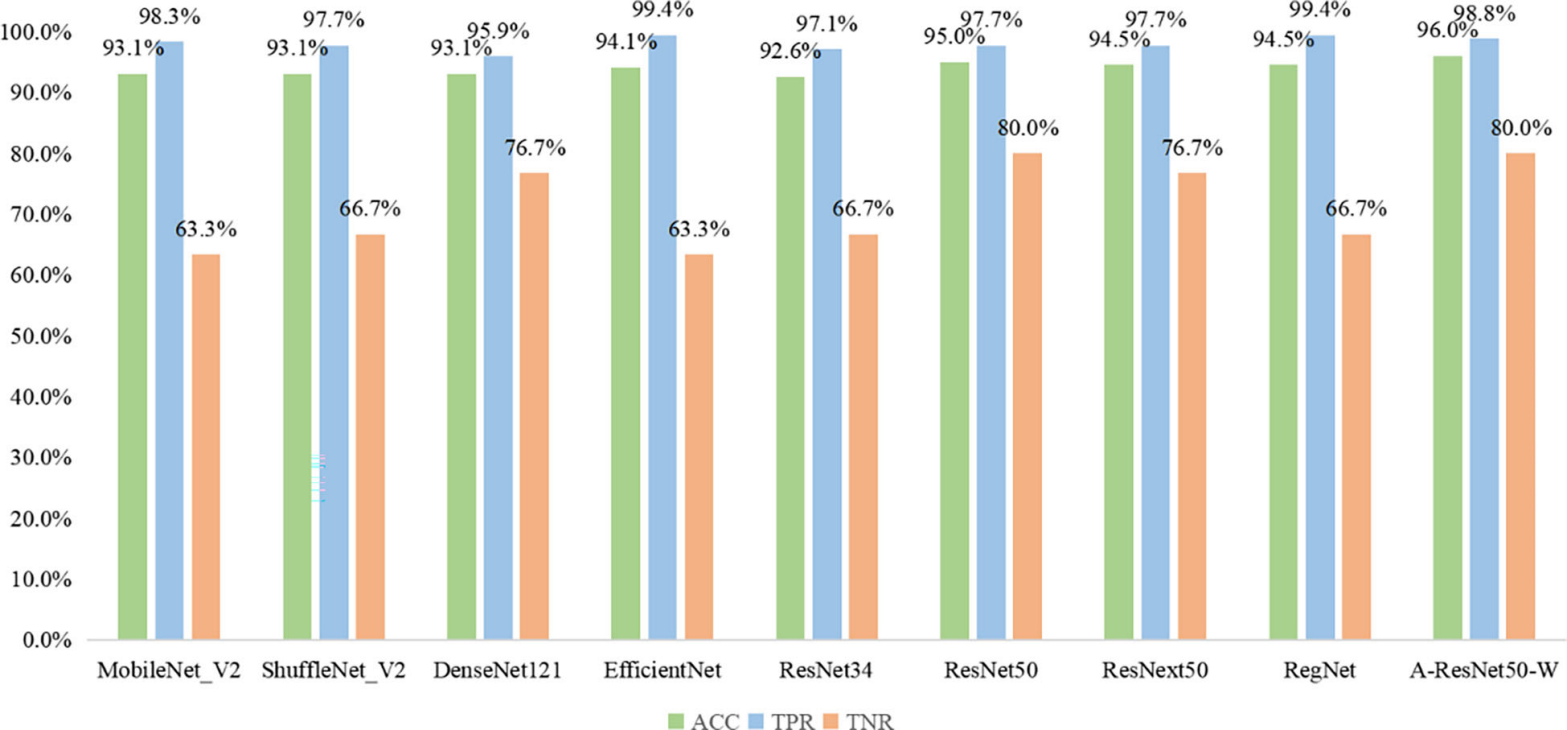
ROC curves of the proposed model vs. radiologists in the testing set.

The results obtained by the proposed method were compared with those predicted by other eight start-of-the-art neural networks, namely MobileNet_V2 (37), ShuffleNet_V2 (38), DenseNet121 (39), EfficientNet (40), ResNet34 (31), ResNet50 (31), ResNext50 (41) and RegNet (42). As shown in Fig 8, the MobileNet_V2, ShuffleNet_V2, DenseNet121, EfficientNet, ResNet34, ResNet50, ResNext50, RegNet, and the proposed method provided the following results: ACC of 93.1, 93.1, 93.1, 94.1, 92.6, 95, 94.5, 94.5 and 96%; TPR of 98.3, 97.7, 95.9, 99.4, 97.1, 97.7, 97.7, 99.4, and 98.8%; TNR of 63.3, 66.7, 76.7, 63.3, 66.7, 80.0, 76.7, 66.7, and 80%, respectively. Thus, the proposed classification network outperformed all eight state-of-the-art ones in ACC, seven in TNR, and six in TPR. Among the alternative networks, ResNet50 had the highest TNR value of 80.0%, which was equal to that of the proposed network, while its ACC and TPR values (95.0 and 97.7%) did not reach the proposed network’s ACC=96% and TPR=98.8%. Noteworthy is that EfficientNet and RegNet had higher TPR values (99.4%) than the proposed network (98.8%) but lower TNR values (63.3 and 66.7%) versus the proposed one (80%). Given their worse ACC parameters (93.1 and 94.5% versus 96%), the proposed method had the best integrated predictive performance among the other eight networks under study.

Besides the comparison with deep learning methods, the proposed method’s predictions were compared against those of three radiologists with six to ten years of clinical experience in LNM diagnosis. **Table 4** shows the ACC, TPR, TNR, and AUC values provided in the testing set by the proposed method and three experienced radiologists. The corresponding ROC curves are plotted in **Figure 8**. It can be found that the area under the corresponding curves of A-ResNet50-W (in orange) is larger than that of the other three radiologists, which intuitively indicates that the classification method proposed in this paper has better diagnostic performance for LNMs on CT images than that of radiologists.

**Table 4:** Classification performance of different structures.

	ACC, %	TPR, %	TNR, %
ResNet50	95.0	97.7	80.0
A-ResNet50	95.5	98.8	76.0
ResNet50-W	95.5	97.7	83.3
A-ResNet50-W	96.0	98.8	80.0

As shown in **Table 5**, the proposed method achieved an AUC of 0.894, which significantly exceeded the estimates of the three radiologists. Specifically, the averaged AUC value of three radiologists was 0.731, with the lowest AUC estimate of 0.695 and the highest of 0.779. By TPR and TNR parameters, the diagnostic performance of the proposed method surpassed that of three experienced radiologists. The averaged TNR value of three radiologists was 68.9% (21/30) versus (80.0%, 24/30) of the proposed A-ResNet50-W network. The averaged TPR of three radiologists reached 78.1%, which is much lower than that of A-ResNet50-W (98.8%). Moreover, compared to the ACC of radiologists, that of A-ResNet50-W was improved by 19.3% (96.0% vs. 76.7%). The above findings indicate that proposed method had a significantly improved diagnostic performance of cervical LNM in CT images, as compared to that of three experienced radiologists.

**Table 5:** Classification performance of the proposed method and three radiologists in the testing set.

	ACC, %	TPR, %	TNR, %	AUC
Radiologist1	75.2 (152/202)	76.2 (131/172)	70.0 (21/30)	0.719
Radiologist2	76.2 (154/202)	79.1 (136/172)	60.0 (18/30)	0.695
Radiologist3	78.7 (159/202)	79.1 (136/172)	76.7 (23/30)	0.779
Averaged values of the three radiologists	76.7 (155/202)	78.1 (134/172)	68.9 (21/30)	0.731
A-ResNet50-W	96.0 (194/202)	98.8 (170/172)	80.0 (24/30)	0.894

### 3.3 Experimental setup

The detection network was trained with a stochastic gradient descent *via* an Nvidia Titan XP graphics card with graphic processing units (GPUs). The maximum learning iteration, learning rate, decay rate, and gamma were set at 500, 0.001, 0.0005, and 0.33, respectively. The detection performance was evaluated by average precision (denoted as AP_50_) with the threshold of the Intersection over Union (IoU) of 0.5.

The classification network was trained with stochastic gradient descent on an Nvidia Titan XP graphics card with graphic processing units (GPUs). The maximum learning iteration, learning rate, decay rate, and gamma were set at 1000, 0.001, 0.0005, and 0.1, respectively. The classification performance was evaluated by accuracy (ACC), true positive rate (TPR), true negative rate (TNR), the receiver operating characteristic (ROC) curve, and the area under the curve (AUC) parameters.

## 4 Discussion & conclusions

**Figure 9:**
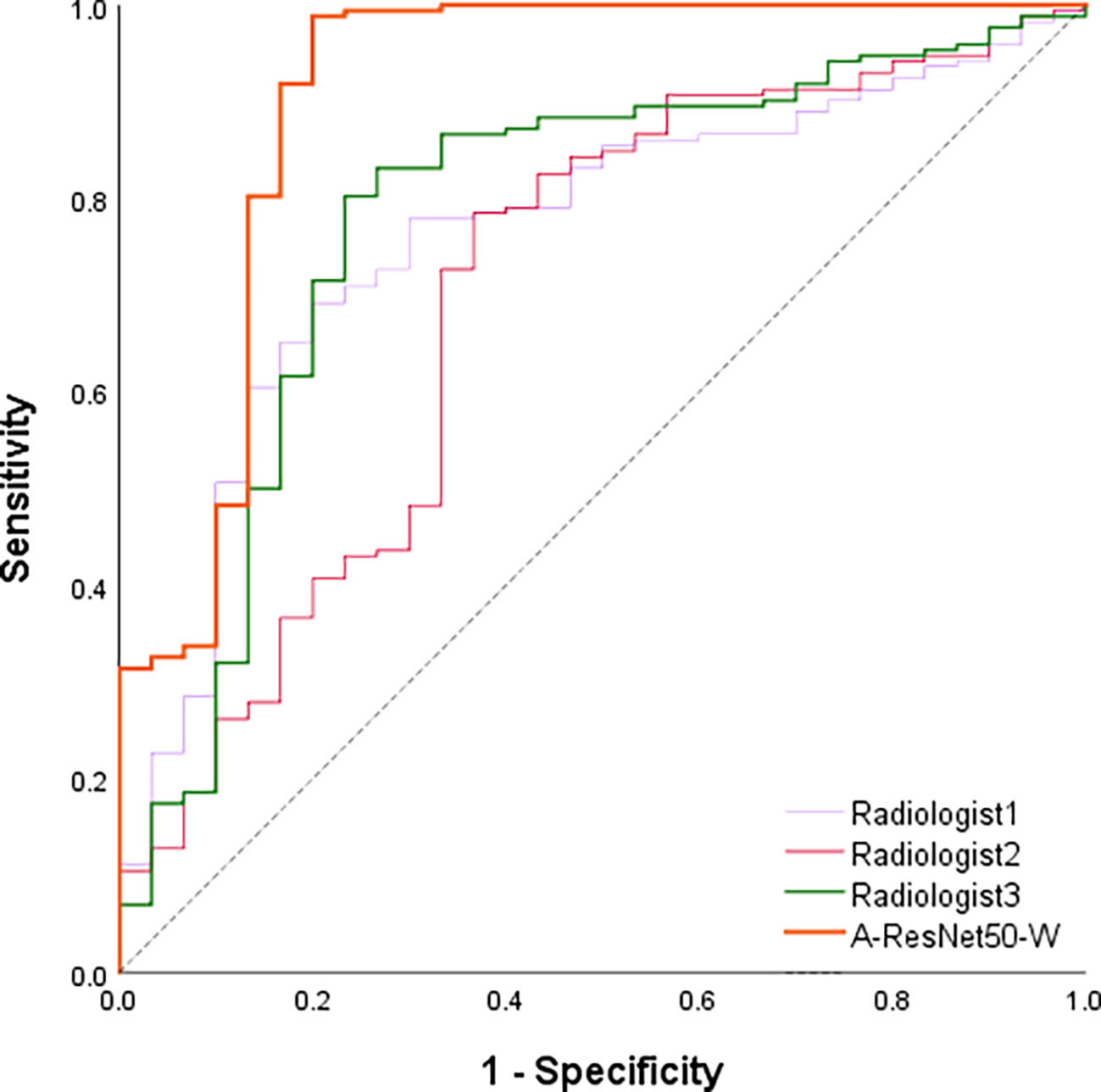
The classification performance of cervical LNM using CT images for different networks.

This study presents a deep learning-based model to diagnose cervical LNM with thyroid carcinoma using preoperative CT images. To the best of the authors’ knowledge, this was the first attempt to apply small object detection to find LNs in CT images. Compared with the other three state-of-the-art detection networks, the proposed network achieved the best AP_50_ parameter, as shown in **Table 3**. Besides, this study was the first to apply the attention mechanism to the classification of cervical LNM in CT images to allow the model to learn more important texture information. The comparative analysis of the proposed network’s accuracy with those of several state-of-the-art classification networks proved that the proposed model outperformed the available algorithms in the classification of cervical LNM in CT images, as shown in **Figure 9**. According to the model’s visualization results listed in **Table 4**, its ACC, TPR, TNR, and AUC parameters in diagnosing cervical LNMs exceeded the respective averaged values of three experienced radiologists. Therefore, the proposed method is considered instrumental in a clinical setting to diagnose cervical LNM with thyroid carcinoma using preoperative CT images.

The proposed CAD method implementation can mitigate several clinical problems. Firstly, the CAD method can reduce the radiologist’s workload by reducing the inherent dependence of the diagnostic process on radiologists. Secondly, diagnostic results of different radiologists on the same CT images may be biased by the human factor, while the application of quantitative criteria in the CAD method ensures accurate and consistent results, which would potentially eliminate the obstacle of inter-observer variability (43). Thirdly, the CAD method has good diagnostic performance and can be used as an auxiliary tool to help radiologists make clinical diagnosis for LNM. Finally, the CAD method may potentially reduce the frequency of unnecessary FNAs for benign LNs. In the future, we are also going to use the breast cancer dataset for testing our proposed network. The experimental results would help us to improve the detection and classification networks we proposed.

However, this study has several limitations. Firstly, relatively few CT images of LNM were collected in this paper due to the limitation of time. Secondly, the experiment was only conducted on the CT dataset from the Second Affiliated Hospital of the Zhejiang University, without multi-center verification. So the results can be systematically biased. Thirdly, the ROIs of LNM was labeled by the radiologist, so the results highly depended on the radiologist’s experience.

In conclusion, a deep-learning-based CAD framework guided by the CT dataset analysis, consisting of an improved Faster R-CNN and the A-ResNet50-W classification network, was proposed for lymph node (LN) detection and classification in CT images in this study. The proposed method outperformed three state-of-the-art detection and classification networks and three experienced radiologists in terms of both detection and classification accuracy. The proposed CAD method can be used as a reliable second opinion for radiologists to help them avoid misdiagnosis due to work overload. Furthermore, it can give helpful suggestions for junior radiologists with limited clinical experience. The follow-up studies envision collecting more CT images of cervical LNM from multiple hospitals to make the CAD method more robust. Besides, unsupervised or weakly supervised learning should be suggested for model training to reduce the burden of data annotation. Finally, future research can consider adding radiologists’ experience and domain knowledge into the deep-learning based CAD method to make it more clinically significant.

## Data availability statement

The raw data supporting the conclusions of this article will be made available by the authors, without undue reservation.

## Author contributions

TW: substantially contributed to the conception and design of the work, and data processing, and doing experiments and the writing of the manuscript, and be accountable for the manuscript’s contents. DY: substantially contributed to the conception and design of the work, and data processing, and doing experiments and the writing of the manuscript, and approved the final version of the manuscript. ZL: substantially contributed to CT data collection. LX, CL, HX, MF: substantially contributed to CT data labling. ZZ: substantially contributed to the conception and design of the work, and investigation, and the revision of the manuscript, and approved the final version of the manuscript, and be accountable for the manuscript’s contents. YW: substantially contributed to writing the final version of the manuscript. All authors contributed to the article and approved the submitted version.
